# Fibromatosis Colli on Point of Care Ultrasound (POCUS): A Case Report

**DOI:** 10.24908/pocusj.v10i01.17787

**Published:** 2025-04-15

**Authors:** Jeff Yang, Christine Rizkalla

**Affiliations:** 1 Department of Emergency Medicine, Maimonides Medical Center, Brooklyn, NY, USA; 2 Department of Pediatric Emergency Medicine, Maimonides Medical Center, Brooklyn, NY, USA

**Keywords:** pediatric, neck mass, point of care ultrasound, musculoskeletal ultrasound

## Abstract

Fibromatosis colli is a rare benign fibrosis of the sternocleidomastoid (SCM) muscle which manifests clinically as congenital muscular torticollis, or an ipsilateral head tilt with contralateral turning of the neck. Though benign, care must be taken not to miss other etiologies with similar clinical presentations, such as malignancies or abscesses. Point of care ultrasound (POCUS) offers a rapid, low-cost, and accessible option for adjunct imaging. In this case report, a 26-day-old boy presented to the pediatric emergency department for evaluation of a nontender, firm left-sided neck mass with preferential turning of his head to the right. POCUS showed a noncompressible, homogenous, well-defined mass in the left SCM without increased vascularity consistent with fibromatosis colli. The patient was discharged from the emergency department with outpatient follow-up. His follow-up course required no further imaging or testing, and the patient's symptoms resolved with physical therapy.

## Introduction

Torticollis, also known as wryneck, is a clinical finding among infants that manifests as a lateral twisting of the neck. Although its precise etiology is still unknown, it is hypothesized that malposition of the neck during intra-uterine pregnancy or complicated delivery causes localized ischemia or trauma within the sternocleidomastoid muscle (SCM). The resulting fibrosis, termed fibromatosis colli, causes contraction of the SCM, which tilts the head towards the affected side and turns the chin toward the contralateral side. Histologically, fibromatosis colli will present with diffuse infiltration or replacement of muscle tissue with fibrous scar tissue [1, 2].

Prevalence of fibromatosis colli is estimated to be 0.3-1.9% of all births, with higher incidence among infants with prolonged or traumatic births [3]. In 6-20% of cases, there will also be associated musculoskeletal anomalies, including developmental dysplasia of the hip (DDH) [4]. Cases typically present 2-4 weeks after birth. For unclear reasons, the right SCM is more commonly involved and accounts for up to 75% of cases, although left-sided and bilateral involvement have also been reported [5, 6]. If unmanaged, plagiocephaly may result, due to infants favoring sleep with the affected side down [7, 8]. Initial conservative management with range-of-motion exercises and other forms of physical therapy is effective in up to 95% of cases [4]. If conservative management fails, botulinum injections or surgical intervention may be considered for persistent torticollis beyond 11 months [7].

## Case Presentation

A 26-day-old boy born at 39 weeks, 5 days via normal spontaneous vaginal delivery was referred to the pediatric emergency department by his pediatrician for evaluation of a left-sided neck mass. His mother first noticed this mass the day of presentation and stated he has preferentially turned his head towards his right since birth. His birth record noted he was born large for gestational age but experienced an uncomplicated birth. He was otherwise healthy, including no prior concerns about his activity or diet.

**Figure 1.**
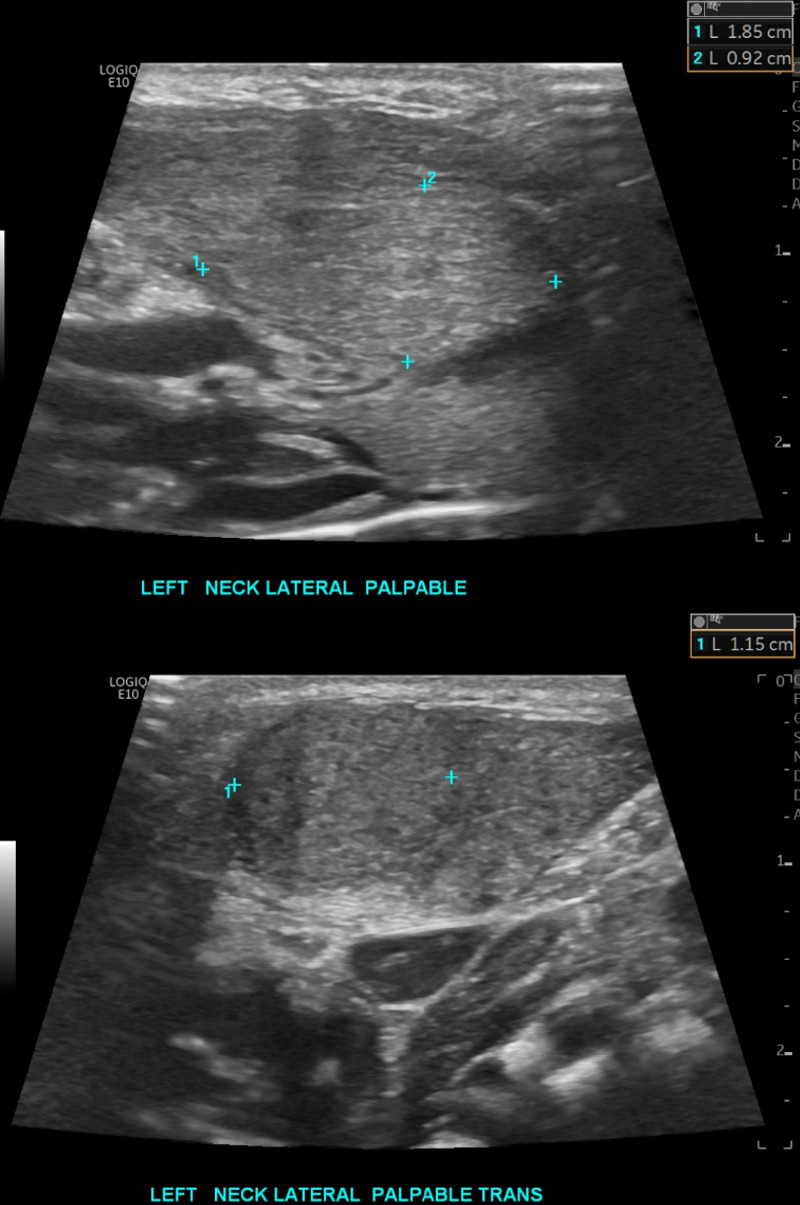
Longitudinal and transverse views demonstrate a homogenous mass in the left sternocleidomastoid (SCM) with well-defined borders and no extension beyond the SCM.

**Figure 2.**
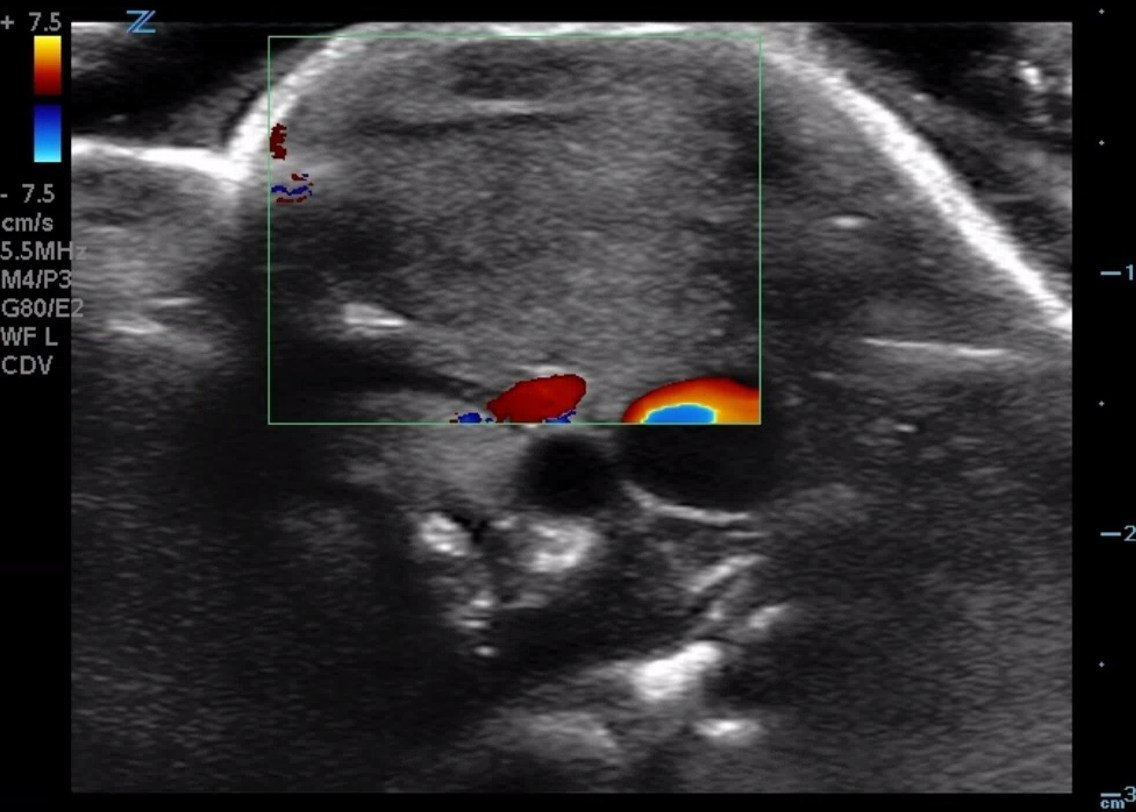
Color Doppler view demonstrates no enhanced vascularity within the mass.

On examination, the boy appeared vigorous, alert, and well overall. Recorded vital signs included a temperature of 98.4°F, heart rate of 127 beats per minute, respiratory rate of 40 breaths per minute, and saturating 99% on room air. His neck exam was remarkable for a 2 cm x 1.5 cm firm, mobile, non-tender mass along the left SCM, leftward head tilt, and slightly limited range of motion of his neck when turning towards stimuli to the left. Bloodwork was notable for no leukocytosis, no elevated C-reactive protein, and no elevated erythrocyte sedimentation rate (Table 1). A point of care ultrasound (POCUS) was performed to better visualize the underlying anatomy of the mass. This revealed a discrete, homogenous, solid mass contained within the left SCM with similar echotexture to the rest of the muscle. The exam and imaging findings were consistent with fibromatosis colli, and the patient was discharged from the emergency department with referral for pediatric surgery clinic follow-up (Figures 1-2, Video S1). Over the next four weeks, the patient was referred to physical therapy and his mother was instructed to gently turn the boy's head leftwards and massage the mass several times a day. At his next follow-up, his exam was notable for resolution of his neck mass and return of full range of motion of the neck.

**Table 1.** Observed bloodwork values are within normal limits.

Laboratory Test	Observed Laboratory Value	Normal Reference Range
WBC	11.0 x 103/µL	9.0-30.0 x 103/µL
RBC	5.34 x 106/µL	3.0-5.4 x 106/µL
Hgb	17.7 g/dL	10.0-18.0 g/dL
Hct	50.8 %	31.0-55.0%
MCV	95.1 fL	85.0-123.0 fL
MCH	33.1 pg	28.0-40.0 pg
MCHC	34.8 g/dL	29.0-37.0 g/dL
RDW	14.8%	11.4-16.4%
Platelet Count	443 x 103/µL	150-450 x 103/µL
TSH	2.14 mIU/L	0.39-4.08 mIU/L
C reactive Protein	< 0.020 mg/dL	0.0-0.3 mg/dL
ESR	8 mm/h	0-15 mm/h

## Discussion

We describe a case of suspected fibromatosis colli based on clinical findings, that were further supported by POCUS of the neck. Although fibromatosis colli is a clinical diagnosis, adjunct lab work and imaging may be performed to further differentiate it from other head and neck pathologies that can present similarly, including vascular or solid tumors, abscesses, lymphadenopathy, and embryonal remnants. In this case, our patient presented with certain clinical features typical of fibromatosis colli, including a unilateral, nontender, firm neck mass with preferential ipsilateral head tilt and contralateral head turn, and absence of other symptoms. However, this patient had no history of birth trauma, and the mass was not right-sided. The infant's vital signs were within normal limits for his age, and his appearance, bloodwork results, and physical exam lowered suspicion for infectious etiology or thyroid mass.

POCUS was utilized to support the diagnosis of fibromatosis colli. Due to its availability, low cost, and lack of exposure to ionizing radiation, ultrasound is the imaging modality of choice in suspected fibromatosis colli. When conducting POCUS of the neck, a high frequency linear probe should be utilized since areas of interest are superficial. The patient should be scanned in an upright or supine position, with the neck turned slightly away from the side being imaged. Images should be obtained in transverse and longitudinal views. Additional considerations include obtaining video clips to assess for compressibility and contralateral imaging to assess for symmetry. Color or power Doppler may be obtained to assess for vascularity.

Fibromatosis colli on POCUS features a focal or diffuse hypertrophy of the SCM with variable echogenicity compared to normal muscle [4, 9]. Rarely, this enlargement may contain calcified foci that suggest prior hemorrhage [9]. Importantly, there should be no extra muscular involvement, no lymphadenopathy, and no irregular margins [5, 9]. These features are consistent with the ultrasound findings in our patient, which showed a focal homogenous 1 cm x 1 cm x 2 cm mass contained within the left SCM.

POCUS is also useful in differentiating fibromatosis colli from other causes of neck masses including rhabdomyosarcoma, neuroblastoma, lymphoma, branchial cleft cysts, hygromas, abscesses, and lymphadenopathy [4, 6, 9]. Neuroblastomas will show internal calcifications and encasement of vessels, often with bulky lymphadenopathy [10]. Rhabdomyosarcomas and other sarcomas will have heterogenous, irregular masses [11]. Structures such as dermoid cyst, teratomas, or thyroglossal duct cysts will appear midline. Infantile hemangiomas will have increased Doppler signal with high vessel density [12]. Similarly, lymphadenopathy will also have increased vascularity and will be discrete from the SCM [9]. Inconclusive POCUS examination may be followed up with CT or magnetic resonance imaging (MRI), and rarely with fine needle aspiration or excisional tissue biopsy [2].

The use of POCUS in an emergency department setting reduces unnecessary testing and leads to a reduction in the average length of stay. In a retrospective cohort study that imaged pediatric neck masses in the emergency department, POCUS was compared to ultrasonography studies performed and interpreted by the radiology department. There was an interobserver agreement of k=0.87 between POCUS images obtained and interpreted by ultrasound fellowship trained pediatric emergency department attendings compared to final ultrasound interpretations from imaging done by the radiology department. At the same time, emergency department length of stays were reduced by an average of 90-120 minutes when POCUS was utilized [13–15].

Although fibromatosis colli is often a benign condition, it may prompt further screening for DDH. These two conditions are coexistent in up to 20% of cases [16]. This may be reflective of a common etiology, such as conditions leading to malpositioning in utero or a traumatic birth [17]. Multiple guidelines recommend universal screening for DDH with physical exam in all newborns and dedicated imaging with ultrasound or radiography in select infants with risk factors [18, 19]. Although these guidelines do not specify fibromatosis colli as one of these risk factors, several studies suggest that there is sufficient association between fibromatosis colli and DDH to warrant universal imaging to screen for DDH in this patient group [16, 17].

## Conclusion

In this case report, POCUS was utilized as a rapid, accessible, non-invasive diagnostic modality for a 26-day-old infant presenting to the emergency department with an unspecified left-sided neck mass. In this instance, it reaffirmed the clinical suspicion of fibromatosis colli while also excluding other causes of neck masses, including hemangioma, tumors, lymph nodes, abscess, or infection. In this case, as well as most cases of fibromatosis colli, resolution was achieved with conservative treatment alone, including physical therapy and passive stretching. Although this condition is often benign, it is important to diagnose to prevent development of plagiocephaly. It may also prompt more careful screening for DDH. Physical therapy alone is usually sufficient to resolve the torticollis. Rarely, surgical correction is necessary for severe cases or for cases refractory to conservative management.


